# Morphological Characteristics of the Nasopalatine Canal and the Relationship with the Anterior Maxillary Bone—A Cone Beam Computed Tomography Study

**DOI:** 10.3390/diagnostics11050915

**Published:** 2021-05-20

**Authors:** Pavle Milanovic, Dragica Selakovic, Milica Vasiljevic, Nemanja U. Jovicic, Dragan Milovanović, Miroslav Vasovic, Gvozden Rosic

**Affiliations:** 1 Department of Dentistry, Faculty of Medical Sciences, University of Kragujevac, 34000 Kragujevac, Serbia; pavle11@yahoo.com (P.M.); milicavaska13@gmail.com (M.V.); 2 Department of Physiology, Faculty of Medical Sciences, University of Kragujevac, 34000 Kragujevac, Serbia; dragica984@gmail.com; 3 Department of Histology and Embryology, Faculty of Medical Sciences, University of Kragujevac, 34000 Kragujevac, Serbia; nemanjajovicic.kg@gmail.com; 4 Clinical Pharmacology Department, Clinical Centre Kragujevac, 34000 Kragujevac, Serbia; piki@medf.kg.ac.rs; 5 Department of Pharmacology and Toxicology, Faculty of Medical Sciences, University of Kragujevac, 34000 Kragujevac, Serbia

**Keywords:** nasopalatine canal—NPC, anterior maxilla, cone beam computed tomography—CBCT, morphometric analysis, linear regression

## Abstract

The aim of this study was to evaluate the interconnection between the type of nasopalatine canal (NPC) and morphometric characteristics of the anterior maxilla. The investigation involved 113 subjects, and the morphometric parameters were obtained using cone beam computed tomography (CBCT). NPC shapes were classified into four types: banana-, hourglass-, cylindrical-, and funnel-shaped (distribution of approximately 9, 25, 31, and 35%, respectively). The analysis revealed that the NPC shape was significantly connected with the morphometric properties of anterior maxilla horizontal dimensions. In general, a banana-shaped NPC was accompanied with an overall reduction in anterior maxilla diameters when compared to other NPC shapes, with no significant difference between the other three NPC shapes. Furthermore, the morphometric characteristics that depend on NPC shape at the sagittal cross-section were significantly correlated with diameters of the incisive foramen, nasal foramen, and NPC length. According to the results of our study, it seems that the presented analyses of morphometric data may allow useful insight into the algorithms of various interconnections between the measures obtained in the region of the anterior maxilla, which could be of interest for a time rationale approach when planning some surgical procedures, such as immediate dental implant placement planning.

## 1. Introduction

In order to achieve progress in the planning of various surgical procedures in dentistry and surgery (both oral and maxillofacial) that employ the interventions in the anterior maxilla, the knowledge of morphometric characteristics of the nasopalatine canal (NPC) seems to be of crucial importance. These data substantially improve the prediction and outcome for diagnostic, anesthesia, and surgical procedures, which include dental implant placement, extraction of impacted teeth, enucleation of NPC cysts, or surgical apicoectomy [1]. As is already known, the NPC is located in the midline, posterior to the maxillary incisors, while its palatal opening (incisive foramen) is located underneath the mucosal incisive papilla. The content of the NPC includes the nasopalatine nerve, the terminal branches of the nasopalatine artery, and the veins, forming a neurovascular bundle surrounded by connective tissue, fat, and seromucous glands [2]. A huge improvement in better understanding the NPC occurred simultaneously with the rapid development and widespread use of 3D cone beam computed tomography (CBCT). This easily accessible, easy to handle, and low radiation dose method provides reliable 3D information about the shape, location, and dimensions of the NPC, as well as its anatomical variations [3]. According to this diagnostic procedure, Mardinegar et al. [4] and Guncu et al. considered four different types of sagittal NPC shapes (hourglass, funnel, banana, and cylindrical) [5], while Liang et al. defined two shapes of the NPC (conical and cylindrical) [2]. However, Etoz and collaborators in 2014 offered the classification of sagittal NPC shapes into six groups (tree branch, cylindrical, banana-like, funnel-like, cone-like, and hourglass [6]), while Safi et al. classified sagittal NPC shapes as cylindrical, funnel-shaped, hourglass, and spindle [7]. The other studies based on CBCT have also shown other variations of the shape and dimensions of the NPC and buccal bone plate [8,9,10]. Furthermore, it should be noted that there is significant variability of the shapes and dimensions of the NPC, depending on the evaluated population. Accordingly, in the Turkish population, the most exposed sagittal NPC shape was hourglass [6], while, in the west Iran subpopulation, it was cylindrical [11]. Furthermore, in the Indian population, the dominant NPC shape was cylindrical [12], while in the Sri Lankan population, the funnel is the most common variation [13]. Interestingly, neither of the mentioned reports confirmed gender and age differences by means of the NPC shape [6,7]. In contrast, the bone resorption patterns were significantly affected by the NPC shape [14,15,16].

The clinical importance of NPC variations has been confirmed in numerous studies. It has been reported that the increase in NPC dimensions and the presence of the bulging sign may be associated with dentoalveolar injury [17]. Furthermore, it has been confirmed that the location and extension of the nasopalatine canal need to be evaluated before the implant placement [18]. Since a safe distance between critical anatomical landmarks and a dental implant is necessary for successful (and stable) implant treatment [19], the labiopalatal width has been included in the checklist for diagnostic parameters before the implant placement [20]. Furthermore, as the implant placement into the NPC occasionally includes various complications, such as bleeding during the operation, postoperative short-term sensory disorder, non-osseointegration of the implant, and the formation of an NPC cyst [21], more precise insight into the anatomical variations of the NPC seems to be essential for avoiding at least some injuries [22]. Taking into account all of the facts mentioned above, it is not surprising that the American Academy of Oral and Maxillofacial Radiology recommends using cross-sectional imaging for the assessment of all dental implant sites, and that CBCT is the imaging modality of choice for gaining information [23].

The aim of this study was to allow better insight into variations of the morphometric characteristics of the NPC. Furthermore, the practical implication of this investigation may be found in the potential application of the observed connections between the morphometric data that may be a solid background for clinical trials that rely on this diagnostic procedure.

## 2. Materials and Methods

### 2.1. Patient Selection

This was a retrospective, quantitative study with CBCT images from patients who came to the Department of Dentistry of Faculty of Medical Sciences, University of Kragujevac, Serbia, during the period from April 2018 to October 2020. The study was carried out in abidance with the Declaration of Helsinki. The study was approved by an institutional review board of the Faculty of Medical Sciences, University of Kragujevac (approval ID 01-4376). Patients came to the Department of Dentistry for different therapeutic reasons: oral surgery, dental implants, prosthetics, periodontology, orthodontic, endodontics, and others. The inclusion criteria were as follows: age over 18, presence of both lateral and central maxillary incisors, and the consent to use clinical data for scientific purposes. The excluding criteria were predefined as low image quality, patients with systemic diseases, such as hyperparathyroidism, Paget’s disease, osteoporosis, and maxillary osteonecrosis, and pathology in the area of the NPC, such as nasopalatine duct cyst, tumors, impacted teeth, cleft lips, periodontal diseases (radiographic bone level from cementoenamel junction > 3 mm), and patients with orthodontic braces and other metal restoration in the area of the maxillary lateral and central incisors. Furthermore, patients with dental implants, bone grafting in the region of the anterior maxilla, and spaced dentition were excluded from trial. According to these criteria, the total number of subjects enrolled in this study was 113 (63 male and 50 female, 45.20 ± 2.14 and 41.06 ± 1.96 average age, respectively).

### 2.2. Characteristics of Imaging Device and Software for Evaluation of Images

The scans were obtained using an Orthophos XG 3D device (Sirona Dental Systems GmbH, Bensheim, Germany), with three-dimensional settings for recording, VOL1 HD (85 kV/6 mA, exposure time—14.3 s) or VOL2 HD (85 kV/10 mA, exposure time—5.0 s), and a voxel size of 160 μm or 100 μm, respectively. The Frankfort horizontal plane was perpendicular to the floor for all scans. For all CBCT images, the field of view was 8 × 8 cm.

CBCT images were analyzed using GALAXIS software v1.9.4 (Sirona Dental Systems GmbH, Bensheim, Germany). Observations were conducted using a 23-inch Philips LED monitor with a resolution of 1920 × 1080 pixels, in a room with dim lighting. Brightness and contrast were adjusted by using software.

### 2.3. Nasopalatine Canal Morphology and Dimensions of Different Sections at Predefined Levels of the Nasopalatine Canal

**Figure 1:**
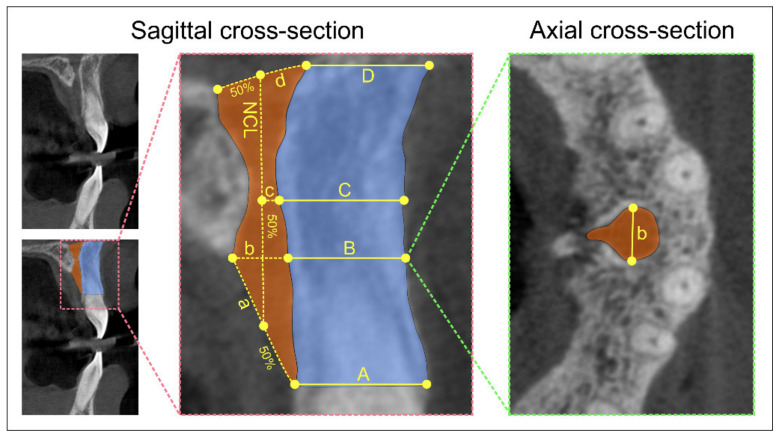
CBCT image and landmarks. Sagittal cross-section—sagittal CBCT slice (left, upper); slice with marked field of interest (left, bellow); selected landmarks for analyses (right)—(**a**) A-P diameter of the incisive foramen, (**b**) level for M-L measurement of the incisive foramen, (**c**) mid-length of the NPC, (**d**) A-P diameter of the nasal foramen, NCL (NPC length), (**A**) horizontal dimension of the anterior maxilla from the buccal border of incisive foramen to the cortical layer, (**B**) horizontal dimension of the anterior maxilla from the buccal wall of the nasopalatine canal to the cortical layer using a horizontal line from the palatal border of the incisive foramen, (**C**) horizontal dimension of the anterior maxilla from the buccal border at the midpoint level of NCL to the cortical layer, (**D**) horizontal dimension of the anterior maxilla from the buccal border of nasal foramen to the cortical layer; axial cross-section—(**b**) M-L diameter of the incisive foramen.

We evaluated the sagittal shape of the NPC according to the previously established criteria [4,9], and the sagittal cross-section types were classified as banana-, hourglass-, cylindrical-, and funnel-shaped. For all defined sagittal shapes of the NPC, the following parameters were determined (Figure 1) and expressed in mm:

1. Antero-posterior diameter (A-P) of nasal foramen (sagittal cross-section)
2. Canal length (sagittal cross-section)
3. Antero-posterior diameter (A-P) of incisive foramen (sagittal cross-section)
4. Mediolateral diameter (M-L) of incisive foramen (axial cross-section).

### 2.4. Horizontal Dimension of Anterior Maxilla

The horizontal dimension of the anterior maxilla was evaluated at the predefined levels using a sagittal cross-section CBCT slice and expressed in mm (Figure 1):

1. A level: distance between the buccal border of incisive foramen and the cortical layer
2. B level: distance between the buccal wall of the nasopalatine canal and the cortical layer using a horizontal line from the palatal border of the incisive foramen
3. C level: distance between the buccal border at the midpoint level of NCL and the cortical layer
4. D level: distance between the buccal border of nasal foramen and the cortical layer.

Two independent observers who made the measurement were blind to the protocol and showed high inter-rater reliability (Pearson’s *r* = 0.95). The mean value for each parameter was taken for further evaluation.

### 2.5. Statistical Analysis

The data presented herein were expressed as the means ± SEM. The parameters were initially submitted to Levene’s test for homogeneity of variance and to the Shapiro–Wilk test of normality. Comparisons between groups were performed using one-way ANOVA, followed by Scheffe’s post hoc test. Pearson’s coefficient of correlation was used to analyze relationships between parameters, and simple linear regression analyses were performed. A value of *p* < 0.05 was considered to be significant. Statistical analysis was performed with the SPSS version 20.0 statistical package (IBM SPSS Statistics 20, Armonk, NY, USA).

## 3. Results

The mean values for the M-L and A-P diameters of the incisive foramen, the A-P diameter of the nasal foramen, and the NPC length, as presented in Table 1, were calculated along with the mean values of the horizontal dimension of the anterior maxilla at different levels (A–D).

**Figure 2:**
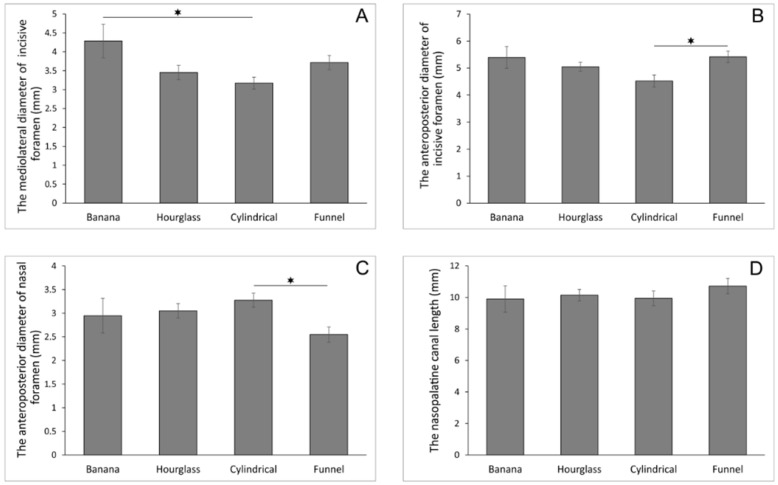
The diameters of the nasopalatine canal according to the nasopalatine canal shape in sagittal cross-section (in mm). (**A**) the mediolateral diameter of incisive foramen; (**B**) the anteroposterior diameter of incisive formane; (**C**) the anteroposterior diameter of nasal foramen; (**D**) the nasopalatine canal length. Values are expressed as the mean ± SEM. * Denotes a significant difference, *p* < 0.05.

The analysis of the NPC shape on sagittal cross-section showed that the banana-shaped canal was present in 10 subjects, the hourglass-shaped canal in 28, and the most frequent shapes of the NPC in this study were the cylindrical- (*n* = 35) and the funnel-shaped that was observed in 40 of the selected patients’ CBCT images. As shown in Figure 2A, the shape of the NPC on sagittal cross-section significantly influenced the M-L diameter of the incisive foramen (F = 3.284, df = 3). Thus, the subjects with the banana-shaped canal showed increased values for this parameter when compared to the cylindrical-shaped ones (*p* < 0.05). In addition, the shape of the NPC on sagittal cross-section significantly affected both the A-P diameter of the incisive foramen and nasal foramen (Figure 2B,C, F = 3.585 and 3.920, respectively), in the opposite manner. Namely, the antero-posterior diameter of the incisive foramen was significantly reduced in the cylindrical-shaped NPC when compared to the funnel-shaped ones (*p* < 0.05), while the A-P diameter of the nasal foramen in the cylindrical-shaped NPC was significantly above the funnel-shaped NPC (*p* < 0.05). Finally, the results presented in Figure 2D confirmed no significant impact of the shape of the NPC on sagittal cross-section of the NPC length (F = 0.634).

In order to estimate the interconnection between the diameter at different sections of the NPC and anterior maxilla dimensions at predefined portions, the linear regression analysis (Table 2) revealed that anterior maxilla horizontal dimension were significantly (negatively) correlated with the M-L diameter of the incisive foramen and the A-P diameter of the nasal foramen at the levels of A, B, and C, while, at the D level of the anterior maxilla horizontal dimension, a significant (positive) correlation was also observed for the NPC length.

**Figure 3:**
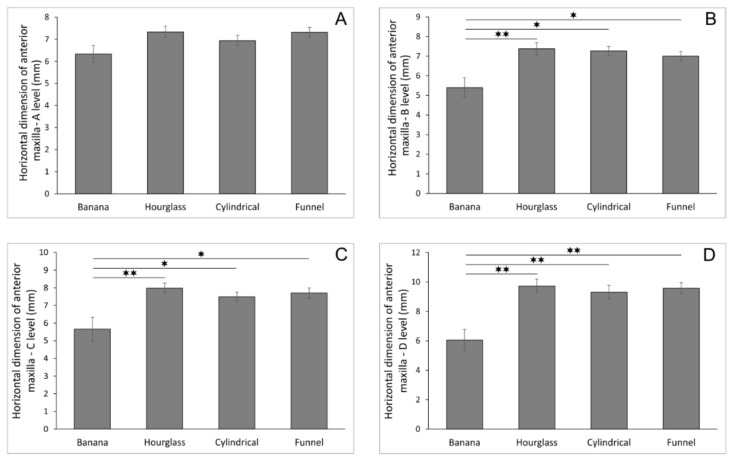
The horizontal dimension of the anterior maxilla at different levels according to the nasopalatine canal shape in sagittal cross-section (in mm). (**A**) horizontal dimension of anterior maxilla—A level; (**B**) horizontal dimension of anterior maxilla—B level; (**C**) horizontal dimension of anterior maxilla—C level; (**D**) horizontal dimension of anterior maxilla—D level. Values are expressed as the mean ± SEM. * Denotes a significant difference of *p* < 0.05, ** denotes a significant difference of *p* < 0.01.

The morphometric analyses also confirmed that the shape of the NPC on sagittal cross-section influenced the horizontal dimension of the anterior maxilla at different levels (A–D levels, Figure 3A–D). Although the NPC shape did not significantly affect the horizontal dimension of the anterior maxilla at level A (Figure 3A, F = 1.790), this diameter was significantly affected by the NPC shape at the other three estimated levels—B, C, and D (Figure 3B–D; F = 4.692, 4.818, and 5.973, respectively). Interestingly, the existence of the banana-shaped NPC was accompanied by the significant reduction at level B (*p* < 0.01 when compared to the hourglass and *p* < 0.05 when compared to the cylindrical and funnel-shaped) and at level C (with equal significance), while the banana-shaped NPC showed the most prominent decline in the horizontal dimension of the anterior maxilla at the D level (*p* < 0.01 when compared to the other three shapes).

Again, the linear regression analysis was used in other to estimate the correlation between the defined diameters (the M-L and A-P diameters of the incisive foramen, A-P diameter of the nasal foramen, and NPC length) and anterior maxilla dimensions at predefined portions depending on the canal shape at the sagittal cross-section. As shown in Table 3, the M-L diameter of the incisive foramen was significantly (negatively) correlated with the A, B, and C levels of the anterior maxilla dimension in the banana-, hourglass- and funnel-shaped canals, with no significant correlation at the anterior maxilla dimension levels for the cylindrical-shaped NPC. In contrast, the only significant (also negative) correlation between the M-L diameter of the incisive foramen and the D level of anterior maxilla was observed in the cylindrical-shaped NPC.

Unlike the confirmation of the impact of NPC shape on the correlation between the M-L diameter of the incisive foramen and anterior maxilla dimensions at different levels for different NPC shapes, the linear regression analysis showed that the only significant (negative) interconnection between the A-P diameter of the incisive foramen and anterior maxilla dimensions was observed in the hourglass-shaped NPC at the B level of the anterior maxilla (Table 4).

A much more pronounced interconnection was confirmed by the linear regression analysis between the A-P diameter of the nasal foramen and anterior maxilla dimensions at different levels for the various shapes of the NPC (Table 5). Interestingly, in the banana- and funnel-shaped canals, the A-P diameter of the nasal foramen was significantly (negatively) correlated with anterior maxilla dimensions at levels A, B, and C. On the other hand, a significant (negative) correlation in the hourglass-shaped NPC was present only between the A-P diameter of the nasal foramen and the C level of the anterior maxilla, while the cylindrical-shaped canal was accompanied only with a significant (also negative) correlation between the antero-posterior diameter of the nasal foramen and the D level of the anterior maxilla.

Finally, the linear regression analysis confirmed that the NPC shape did not significantly influence the mathematical interconnection between the NPC length and anterior maxilla dimensions at any predefined level (Table 6).

## 4. Discussion

The results of this study were obtained to provide the morphometric background for clinically important issues, since the anterior maxilla represents a challenging region from the surgical point of view, which especially refers to immediate implant placement. Dental implants are a widespread and reliable treatment option for restoring function and esthetics in partially and completely edentulous patients [24]. The maxillary anterior region is crucial for the esthetic satisfaction of the patient, and an adequate alveolar ridge dimension must be present. The proximity of the NPC to the roots of the central incisors directs the position of an implant. Anterior maxillary implants represent a significant challenge for the achievement of good esthetics, and their proper restoratively driven position is often impaired due to the proximity and morphology of the NPC. Due to the reasons described above, the anatomical variations of the NPC, such as the shape, dimensions, curvature, and direction, must be considered seriously when planning surgical procedures in the anterior maxilla. Previous studies have shown that there is a significant association between neurovascular bundle injury and implant osseointegration failure [16,25,26]. Kraut and Boiden [27] reported that NPC dimensions were an obstacle to implant placement in approximately 4% of cases, while Alkanderi et al. [24] stated that an NPC perforation rate of 8% could be expected during immediate implant placement in the maxillary central incisor region.

In this study, we also analyzed the morphological characteristics of the NPC on different CBCT sections, as well as its impact on the morphometric properties of the buccal bone in the anterior maxilla. Thus, the starting point in this study was predefined according to the most commonly established classification, based on a cross-sectional view of CBCT scans, into four categories: cylindrical-, funnel-, hourglass-, and banana-shaped canals [4]. Therefore, following these criteria, the results of our study showed that the most frequent shapes of the NPC were funnel-shaped (35.4%) and cylindrical-shaped (31.0%), followed by hourglass-shaped (24.8%) and banana-shaped (8.8%). This is consistent with the results of Fukuda et al. [28] and Mardinger et al. [4], who also had shown that funnel-shaped and cylindrical-shaped canals were predominant in dentate subjects, unlike for edentulous patients [4,29]. On the other hand, Tözüm and coworkers reported that the dental status (presence or absence of teeth) did not influence the NPC shape [30]. In contrast to these studies, Etoz et al. [6] and Sekerci et al. [31] found that the hourglass-shaped NPC was the most common shape. In the study of Gil-Marques and colleagues [9], the banana-shaped NPC was predominant. These variations may be the result of racial, age, and gender differences, but also of the evaluation methodology.

The diameter of the incisive foramen is usually declared as less than 6 mm. When it exceeds 10 mm, the existence of a potential pathological condition (cyst, tumors, etc.) should be considered [32,33]. The evaluation of the sagittal CBCT sections in this study revealed that the average A-P diameter of the incisive foramen and nasal foramen were 5.04 mm and 2.93 mm, respectively. These results are in accordance with previous studies [32,34]. At the same time, Borstein et al. [8] found that the average A-P diameter of the incisive foramen was 4.45 mm, and the A-P diameter of the nasal foramen was 3.48 mm. However, Gönüle al. [35] found slightly larger diameters for both A-P diameters of incisive and nasal foramen (6.31 mm and 3.29 mm, respectively) using multi-detector row computed tomography. In this study, the M-L diameter of the incisive foramen was analyzed at axial sections, and the mean value was 3.53 mm. Although there were only a few studies that evaluated that parameter, Salemi et al. reported that the average value of this parameter was 3.58 mm [36], while some authors presented a slightly smaller diameter [10,12].

Depending on the NPC shape in the sagittal section, we noticed differences in the dimensions of both incisive and nasal foramen. In the banana-shaped canal, the M-L diameter of the incisive foramen was significantly larger than in the cylindrical-shaped canal. A reduced A-P diameter of the incisive foramen can be expected in the case of the cylindrical-shaped canal compared to the funnel-shaped, while the A-P diameter of the nasal foramen in the cylindrical shape was significantly larger. These morphological data could be useful landmark parameters when planning implant placement.

Based on the literature data, NPC length ranges from approximately 8 to 16 mm [7,8,21,37]. The variations in the canal length can be explained by the different measurement methods in previous studies. Regardless of racial differences, the presence or absence of teeth and the measurement technique can be decisive factors in the registered length. NPC length, following the methodology applied in this study, was determined at the level of app. 10.26 mm. This result is in accordance with the pervious study by Thakur et al. [32], who reported that the average NPC length in their study was 10.08 mm, while Mraiwa et al. [33] assessed in their study the length of the NPC at 8.1 mm. On the other hand, the NPC length in our study was not in line with the study by Soumya et al., who reported that the average value was 18.63 mm [12]. The observed difference may be attributed to various populations categories.

Sufficient alveolar ridge width represents a preliminary key indicator for the successful placement of dental implants due to the discrepancies between the diameter of the implants and the horizontal dimension of the alveolar ridge [38]. The horizontal bone dimension of the alveolar ridge in the anterior maxilla in this study was measured on sagittal sections anterior to the NPC at four consecutive levels, as shown in Figure 1, which is not the standard procedure according to the literature data. Namely, while some studies included an evaluation of buccal bone width at the three horizontal levels (distance between the anterior border of the incisive foramen and facial aspect of the buccal bone wall, distance between the anterior border of the NPC and facial aspect of the buccal bone wall using a horizontal line from the palatal border of the incisive foramen, and distance from the buccal border in the middle of the nasopalatine canal to the facial aspect of the buccal bone wall) [8], other investigators that analyzed alveolar ridge width also chose three levels, but at different sections [9]. However, our study offered the morphometric analysis of the alveolar ridge at four distinctive levels, like the ones previously reported by Salemi and coworkers [36], in order to fulfill the range of morphometric properties that can be more useful for clinical practice. On average, the largest dimension was recorded in the apical part of the canal (D level—9.22 mm), and the smallest at the level of the palatal border of the incisive foramen (B level—7.03 mm), which is in line with the results presented by Güncü et al., who recorded horizontal bone dimensions at three different levels (crestal, medial, and apical), where the average dimension of the buccal bone was also largest at the apical level (9.84 mm) [5]. In the study of López et al., similar results were reported for the average horizontal bone dimensions in dentate patients [39].

Alveolar bone thickness defines the occlusal-gingival position of the implant, and it is vital for long-term soft tissue stability [40]. Estimation of the horizontal alveolar ridge dimension at different levels is very important from the aspect of immediate implant placement, since this dimension is larger in the apical than in the crestal part, to assess whether a more apical implant position would provide greater bone thickness in this critical region for the implant placement [41]. To the best of the author’s knowledge, this is the first study that estimated the interconnection between NPC morphological characteristics and horizontal bone dimensions in the anterior maxilla at various consecutive levels. The results of our study revealed that the increased M-L diameter of the incisive foramen and the A-P diameter of the nasal foramen was accompanied with the smaller horizontal alveolar ridge dimension at all levels.

Morphometric analysis also confirmed that the shape of the NPC on the sagittal section influenced the horizontal bone dimension of the anterior maxilla at different levels. The banana-shaped NPC was accompanied by a significant reduction in alveolar bone dimensions at all levels, except the incisive opening level. Such information may be of potential clinical importance in planning implant therapy, since Alkanderi and coworkers [24] in their study analyzing the incidence of perforation of the NPC during virtual implant placement confirmed that the most perforations occurred in the middle third of the implant. Furthermore, Chan and colleagues [42] stated that if an implant was placed following the axis of its restoration, buccal bone fenestrations would occur in approximately 20% of cases, but most commonly in the apical third of the implant. In the cases of a banana-, hourglass-, or funnel-shaped NPC, the larger M-L diameter of the incisive foramen appeared simultaneously with the reduction in the alveolar bone horizontal dimension at the crestal and middle parts. Cylindrical canals with a larger M-L incisive canal diameter indicated a smaller alveolar ridge dimension at the apical part. Furthermore, in the funnel-shaped canal, a larger A-P diameter of the incisive canal indicates a smaller horizontal bone dimension in the B level.

Interestingly, the results of our study showed that the larger nasal foramen A-P diameter occurred concomitantly with the reduction in the horizontal bone dimension of the anterior maxilla at the levels A, B, and C for banana- and funnel-shaped canals. In contrast, an hourglass type of NPC was accompanied with bone thickness reduction only at the level C, while, in the cylindrical NPC type, the larger nasal foramen appeared simultaneously with a significant decline in the horizontal bone dimension at the level D (apical part of the NPC). These results could be interesting for clinicians, since a cylindrical canal with the larger nasal foramen A-P diameter implies the necessity to reconsider the selection of conical-type implants in order to prevent apical bone perforation, as described in the literature [42,43].

In summary, according to the results of our study, it seems that the presented analyses of morphometric data obtained with highly reliable methodology may allow useful insight into the algorithms of various interconnections between the measures obtained in the region of the anterior maxilla. The repetitiveness of the observed relationships may be suitable to serve for preliminary examination (as an early checkpoint) when planning implant placement, especially for clinicians with less experience, by means of time-consuming reduction. Indeed, the proposed methodology does not pretend to replace individual morphometric analysis for each patient, but it may provide good first-step criteria in choosing relevant parameters. Finally, the future investigations, which will also include bone resorption patterns, as well as specific gender and age groups, will significantly improve the benefits of this approach from the perspective of clinical usefulness.

## Figures and Tables

**Table 1:** The diameters at different sections of the nasopalatine canal and the horizontal dimension of the anterior maxilla (mm).

The Sections of the NPC	Mean ± SEM	The Horizontal Dimension of Anterior Maxilla	Mean ± SEM
The M-L diameter of incisive foramen	3.53 ± 0.11	A level	7.11 ± 0.13
The A-P diameter of incisive foramen	5.04 ± 0.12	B level	7.03 ± 0.15
The A-P diameter of nasal foramen	2.93 ± 0.01	C level	7.52 ± 0.17
The NPC length	10.26 ± 0.25	D level	9.22 ± 0.25

**Table 2:** The correlation between the diameter at different sections of the nasopalatine canal and anterior maxilla dimensions at predefined portions.

The Sections of the NPC	The Levels of Anterior Maxilla Horizontal Dimension			
	A Level	B Level	C Level	D Level
The M-L diameter of incisive foramen	y = −0.2679x + 5.4396 R^2^ = 0.1103 ***p* = 0.0003**	y = −0.5650x + 8.4428 R^2^ = 0.1941 ***p* = 1.04 × 10^−6^**	y = −0.3020x + 5.8057 R^2^ = 0.2319 ***p* = 6.67 × 10^−8^**	y = −0.1070x + 4.5191 R^2^ = 0.0650 ***p* = 0.0064**
The A-P diameter of incisive foramen	y = −0.0344x + 7.2914 R^2^ = 0.0010 *p* = 0.7408	y = −0.1272x + 7.6751 R^2^ = 0.0106 *p* = 0.2784	y = −0.0508x + 7.7840 R^2^ = 0.0013 *p* = 0.7041	y = −0.0150x + 9.2974 R^2^ = 5 × 10^−5^ *p* = 0.9402
The A-P diameter of nasal foramen	y = −0.3100x + 8.0271 R^2^ = 0.0473 ***p* = 0.0207**	y = −0.3233x + 7.9812 R^2^ = 0.0401 ***p* = 0.0333**	y = −0.6940x + 9.5630 R^2^ = 0.1431 ***p* = 3.61 × 10^−5^**	y = −0.8513x + 11.718 R^2^ = 0.0965 ***p* = 0.0008**
The NPC length	y = 0.0063x + 7.0533 R^2^ = 0.0001 *p* = 0.8991	y = 0.0041x + 6.9908 R^2^ = 5 × 10^−5^ *p* = 0.9415	y = 0.0010x + 7.5173 R^2^ = 2 × 10^−6^ *p* = 0.9872	y = 0.2360x + 6.7993 R^2^ = 0.0554 ***p* = 0.0120**

Significant correlations are bolded.

**Table 3:** The correlation between the M-L diameter of the incisive foramen and anterior maxilla dimensions at predefined portions depending on the canal shape at the sagittal cross-section.

The Correlation between the M-L Diameter of the Incisive Foramen and Anterior Maxilla Dimensions on Different Levels	The NPC Shape at the Sagittal Cross-Section			
	Banana	Hourglass	Cylindrical	Funnel
The M-L diameter of incisive foramen vs. A level	y = −0.5544x + 8.7112 R^2^ = 0.4044 ***p* = 0.0481**	y = −0.7374x + 9.8784 R^2^ = 0.2845 ***p* = 0.0034**	y = 0.0428x + 6.8023 R^2^ = 0.0008 *p* = 0.8721	y = −0.4862x + 9.1267 R^2^ = 0.1759 ***p* = 0.0070**
The M-L diameter of incisive foramen vs. B level	y = −0.8315x + 8.9629 R^2^ = 0.5505 ***p* = 0.0140**	y = −0.8146x + 10.1860 R^2^ = 0.2389 ***p* = 0.0083**	y = 0.1325x + 6.8406 R^2^ = 0.0079 *p* = 0.6106	y = −0.6788x + 9.5250 R^2^ = 0.3035 ***p* = 0.0002**
The M-L diameter of incisive foramen vs. C level	y = −1.0048x + 9.9655 R^2^ = 0.4335 ***p* = 0.0385**	y = −0.7770x + 10.6700 R^2^ = 0.2767 ***p* = 0.0040**	y = −0.3666x + 8.6585 R^2^ = 0.0479 *p* = 0.2065	y = −0.7856x + 10.6210 R^2^ = 0.2541 ***p* = 0.0009**
The M-L diameter of incisive foramen vs. D level	y = 0.0223x + 5.9534 R^2^ = 0.0002 *p* = 0.9704	y = −0.6056x + 11.8200 R^2^ = 0.0579 *p* = 0.2172	y = −1.1468x + 12.9510 R^2^ = 0.1498 ***p* = 0.0216**	y = −0.2214x + 10.4040 R^2^ = 0.0128 *p* = 0.4871

Significant correlations are bolded.

**Table 4:** The correlation between the A-P diameter of the incisive foramen and anterior maxilla dimensions at predefined portions depending on the canal shape at the sagittal cross-section.

The Correlation between the A-P Diameter of the Incisive Foramen and Anterior Maxilla Dimensions on Different Levels	The NPC Shape at the Sagittal Cross-Section			
	Banana	Hourglass	Cylindrical	Funnel
The A-P diameter of incisive foramen vs. A level	y = 0.0561x + 6.0321 R^2^ = 0.0034 *p* = 0.8720	y = −0.2439x + 8.5649 R^2^ = 0.0242 *p* = 0.4288	y = −0.0008x + 6.9415 R^2^ = 5 × 10^−7^ *p* = 0.9966	y = −0.0770x + 7.7374 R^2^ = 0.0056 *p* = 0.6454
The A-P diameter of incisive foramen vs. B level	y = −0.2531x + 6.7647 R^2^ = 0.0424 *p* = 0.5681	y = −0.7213x + 11.0180 R^2^ = 0.1459 ***p* = 0.0448**	y = 0.2314x + 6.2150 R^2^ = 0.0486 *p* = 0.2032	y = −0.1054x + 7.5743 R^2^ = 0.0093 *p* = 0.5528
The A-P diameter of incisive foramen vs. C level	y = 0.1953x + 4.6053 R^2^ = 0.0136 *p* = 0.7482	y = −0.4346x + 10.1830 R^2^ = 0.0674 *p* = 0.1820	y = 0.1094x + 7.0005 R^2^ = 0.0086 *p* = 0.5971	y = −0.0848x + 8.1612 R^2^ = 0.0038 *p* = 0.7062
The A-P diameter of incisive foramen vs. D level	y = 1.0952x + 0.1390 R^2^ = 0.3653 *p* = 0.0642	y = −0.2964x + 11.2260 R^2^ = 0.0108 *p* = 0.5985	y = −0.1947x + 10.1920 R^2^ = 0.0087 *p* = 0.5948	y = 0.0883x + 9.1033 R^2^ = 0.0026 *p* = 0.7548

Significant correlations are bolded.

**Table 5:** The correlation between the A-P diameter of nasal foramen and anterior maxilla dimensions at predefined portions depending on the canal shape at the sagittal cross-section.

The Correlation between the A-P Diameter of Nasal Foramen and Anterior Maxilla Dimensions on Different Levels	The NPC Shape at the Sagittal Cross-Section			
	Banana	Hourglass	Cylindrical	Funnel
The A-P diameter of nasal foramen vs. A level	y = −0.6705x + 8.3104 R^2^ = 0.4050 ***p* = 0.0479**	y = −0.0976x + 7.6306 R^2^ = 0.0033 *p* = 0.7718	y = 0.0802x + 6.6754 R^2^ = 0.0025 *p* = 0.7751	y = −0.5043x + 8.6054 R^2^ = 0.1371 ***p* = 0.0186**
The A-P diameter of nasal foramen vs. B level	y = −0.9056x + 8.0668 R^2^ = 0.4470 ***p* = 0.0345**	y = −0.4522x + 8.7537 R^2^ = 0.0486 *p* = 0.2596	y = 0.1208x + 6.8657 R^2^ = 0.0059 *p* = 0.6607	y = −0.5650x + 8.4428 R^2^ = 0.1523 ***p* = 0.0128**
The A-P diameter of nasal foramen vs. C level	y = −1.3293x + 9.5751 R^2^ = 0.5194 ***p* = 0.0187**	y = −0.8444x + 10.5630 R^2^ = 0.2157 ***p* = 0.0127**	y = −0.2501x + 8.3141 R^2^ = 0.0200 *p* = 0.4181	y = −0.8599x + 9.8926 R^2^ = 0.2204 ***p* = 0.0022**
The A-P diameter of nasal foramen vs. D level	y = −0.2573x + 6.8070 R^2^ = 0.0166 *p* = 0.7228	y = −0.9414x + 12.6010 R^2^ = 0.0924 *p* = 0.1157	y = −1.4884x + 14.1850 R^2^ = 0.2259 ***p* = 0.0039**	y = −0.7158x + 11.4050 R^2^ = 0.0968 *p* = 0.0507

Significant correlations are bolded.

**Table 6:** The correlation between the nasopalatine canal length and anterior maxilla dimensions at predefined portions depending on the canal shape at the sagittal cross-section.

The Correlation between the NPC Length and Anterior Maxilla Dimensions on Different Levels	The NPC Shape at the Sagittal Cross-Section			
	Banana	Hourglass	Cylindrical	Funnel
The NPC length vs. A level	y = 0.2198x + 4.1584 R^2^ = 0.2267 *p* = 0.1642	y = 0.1037x + 6.2811 R^2^ = 0.0208 *p* = 0.4641	y = 0.0689x + 6.2531 R^2^ = 0.0180 *p* = 0.4420	y = −0.1210x + 8.6179 R^2^ = 0.0717 *p* = 0.0947
The NPC length vs. B level	y = 0.3059x + 2.3691 R^2^ = 0.2658 *p* = 0.1271	y = 0.0901x + 6.4601 R^2^ = 0.0108 *p* = 0.5986	y = 0.0921x + 6.3458 R^2^ = 0.0334 *p* = 0.2930	y = −0.1359x + 8.4604 R^2^ = 0.0801 *p* = 0.0768
The NPC length vs. C level	y = 0.1986x + 3.6923 R^2^ = 0.0604 *p* = 0.4937	y = 0.1174x + 6.7960 R^2^ = 0.0234 *p* = 0.4373	y = 0.0300x + 7.1967 R^2^ = 0.0028 *p* = 0.7627	y = −0.1082x + 8.8615 R^2^ = 0.0317 *p* = 0.2717
The NPC length vs. D level	y = −0.0657x + 6.6996 R^2^ = 0.0056 *p* = 0.8366	y = 0.3583x + 6.0940 R^2^ = 0.0750 *p* = 0.1585	y = 0.2601x + 6.7251 R^2^ = 0.0672 *p* = 0.1325	y = 0.2045x + 7.3890 R^2^ = 0.0718 *p* = 0.0946

## Data Availability

Data available on request from authors.
